# Pregnancy, time to pregnancy and obstetric outcomes among female childhood cancer survivors: results of the DCOG LATER-VEVO study

**DOI:** 10.1007/s00432-020-03193-y

**Published:** 2020-03-27

**Authors:** M. van Dijk, F. E. van Leeuwen, A. Overbeek, C. B. Lambalk, M. M. van den Heuvel-Eibrink, W. van Dorp, W. J. Tissing, L. C. Kremer, J. J. Loonen, B. Versluys, D. Bresters, C. M. Ronckers, H. J. van der Pal, C. C. M. Beerendonk, G. J. L. Kaspers, E. van Dulmen-den Broeder, M. H. van den Berg

**Affiliations:** 1 Department of Paediatric Oncology, Emma Children’s Hospital, Amsterdam UMC, Vrije Universiteit Amsterdam, P.O. Box 7057, 1007 MB Amsterdam, The Netherlands; 2 Department of Epidemiology, Netherlands Cancer Institute, Amsterdam, The Netherlands; 3 Department of Obstetrics and Gynaecology, Amsterdam UMC, Vrije Universiteit Amsterdam, Amsterdam, The Netherlands; 4 Princess Máxima Center for Pediatric Oncology, Utrecht, The Netherlands; 5 Department of Gynaecology and Obstetrics, Erasmus MC-University Medical Centre Rotterdam, Rotterdam, The Netherlands; 6 Department of Haematology, Radboud University Medical Center, Nijmegen, The Netherlands; 7 Department of Obstetrics and Gynecology, Radboud University Medical Center, Nijmegen, The Netherlands

**Keywords:** Childhood cancer survivors, Pregnancy rates, Time to pregnancy, Obstetric outcomes

## Abstract

**Purpose:**

To evaluate pregnancy rates, time to pregnancy (TTP) and obstetric outcomes in female childhood cancer survivors (CCSs) and to identify specific diagnosis- and treatment-related factors associated with these outcomes.

**Methods:**

The study is part of the DCOG LATER-VEVO study, a nationwide multicenter cohort study evaluating fertility among long-term Dutch female CCSs. Data were collected by questionnaire. The current study included 1095 CCSs and 812 controls, consisting of sisters of CCSs and a random sample of women from the general population.

**Results:**

Among the subgroup of women who ever had the desire to become pregnant, the chance of becoming pregnant was significantly lower for CCSs than controls (OR 0.5, 95%CI 0.4–0.8). Moreover, TTP was 1.1 times longer for CCSs compared to controls (*p* = 0.09) and was significantly longer in survivors of CNS and renal tumours. Overall, no differences were found between CCSs and controls regarding the probability of ever having had a miscarriage, still birth, or induced abortion. However, CCSs had a significantly increased risk of delivering preterm (OR 2.2, 95%CI 1.3–3.7) and delivering via caesarean section (OR 1.8, 95%CI 1.2–2.6). Treatment with lower abdominal/pelvic radiotherapy was strongly associated with several adverse obstetric outcomes.

**Conclusion:**

CCSs are less likely to have ever been pregnant. Among those who do become pregnant, certain subgroups of CCSs are at increased risk of longer TTP. Moreover, as pregnant CCSs, especially those treated with lower abdominal/pelvic radiotherapy, are more likely to develop various adverse obstetric outcomes, appropriate obstetric care is highly advocated.

**Electronic supplementary material:**

The online version of this article (10.1007/s00432-020-03193-y) contains supplementary material, which is available to authorized users.

## Introduction

Increased survival rates for childhood cancer have resulted in a growing population of childhood cancer survivors (CCSs) (Gatta et al. 2014). However, chemo- and radiotherapy administered during childhood may compromise female reproductive function resulting in infertility, subfertility, or a premature menopause due to a depletion of the ovarian follicle pool (Green et al. 2002, 2009a; Chemaitilly et al. 2006; Sklar et al. 2006; van den Berg 2018). This may put CCSs at increased risk of a longer time to pregnancy (TTP) as seen in ageing women.

Pregnancy or live birth rates have consistently shown to be reduced among female CCSs, particularly after hypothalamic-pituitary or pelvic radiotherapy and exposure to certain alkylating agents (Green et al. 2009b; Chow et al. 2016; Anderson et al. 2018; Magelssen et al. 2008; Armuand et al. 2017; Madanat et al. 2008). However, marital status or the actual desire to conceive, factors known to be independently associated with pregnancy rates, were not always taken into account. In addition, time to first pregnancy was found to be significantly longer in CCSs compared to siblings, particularly after abdominal radiotherapy, but TTP for subsequent pregnancies was not evaluated (Green et al. 2009b). As such, the impact of a possible treatment-related reduced ovarian reserve on TTP for subsequent pregnancies remains unknown.

Female CCSs considering pregnancy also have significant concerns about treatment-related adverse pregnancy outcomes (Schover et al. 1999a, b). Following pelvic or abdominal radiotherapy women are indeed at increased risk of a miscarriage (Green et al. 2002; Reulen et al. 2009; Reulen et al. 2009; Chiarelli et al. 2000; Winther et al. 2008), preterm delivery, or delivering low-birthweight offspring (Green et al. 2002; Signorello et al. 2006). After cranial radiotherapy, however, conflicting results have been described (Green et al. 2002; Reulen et al. 2009; Winther et al. 2008), whereas chemotherapy has not been associated with adverse obstetric outcomes (Green et al. 2002; Reulen et al. 2009; Chiarelli et al. 2000; Signorello et al. 2006). These studies predominantly evaluated pregnancy outcomes related to first pregnancies only. Therefore, results may underestimate the actual risk of adverse pregnancy outcomes following childhood cancer treatment. Moreover, conflicting results have been described regarding the risk of a caesarean section among female CCSs (Reulen et al. 2017; van der Kooi et al. 2018; Lie Fong et al. 2010).

So far, the effect of specific diagnosis- and treatment-related factors on pregnancy rates in those who pursued a pregnancy, as well as on TTP and adverse obstetric outcomes among female CCSs remain largely unknown. In order to adequately counsel survivors regarding family planning and possible obstetric risks, identifying those at increased risk is warranted so that this information can feed into clinical guidelines for patient care. Therefore, the aim of the current study was to evaluate pregnancy rates, TTP and obstetric outcomes for all pregnancies in female CCSs compared to controls, and to investigate diagnosis- and treatment-related factors associated with these outcomes.

## Methods

### Study population

This study was conducted as part of the DCOG LATER-VEVO study, a nationwide, multi-center retrospective cohort study among Dutch female CCSs aiming to evaluate the effects of childhood cancer treatment on fertility. The study design and data collection methods have been described previously (Overbeek et al. 2012). In short, the study population consisted of adult women, treated for cancer before the age of 18 years between 1963 and 2002 who survived for at least five years after diagnosis (*n* = 2237). Sisters from participating CCSs and women from the general population were included as control subjects (further referred to as controls) (van den Berg et al. 2014). All controls had never been diagnosed with cancer, were able to read and speak Dutch, and were also of adult age at time of study inclusion.

### Data collection and outcomes

Data were collected by a questionnaire (de Boer et al. 2005) which addressed, among other items, socio-demographic characteristics, reproductive history, and lifestyle factors. Data included the number of pregnancies, time to each pregnancy (number of months of unprotected sexual intercourse until pregnancy), duration and outcome of each pregnancy (live birth, still birth, miscarriage, or induced abortion), birth weight, and mode of delivery (caesarean section yes/no). Unsuccessful pregnancies ending before gestational week 20 were considered a miscarriage and from gestational week 20 onwards a stillbirth. A live birth before gestational week 37 was considered a preterm delivery. In addition, an infant was defined as small for gestational age (SGA) if the reported birthweight was below the 10th percentile of Dutch reference curves for birthweight by gestational age (Visser et al. 2009). Detailed data on cancer diagnosis and treatment were collected from original medical files.

### Statistical analyses

Differences between socio-demographic characteristics of survivors and controls were analyzed using Chi-square tests, Student *t*-tests, or Mann–Whitney *U* tests, where appropriate.

Pregnancy rates and TTP were evaluated within the subgroup of participants who ever pursued a pregnancy. As such, virgins, as well as women who had never tried to become pregnant in the past, were excluded from these analyses. Also, women who ever unintentionally became pregnant, and reported that this pregnancy was terminated by means of an induced abortion, were not included in order to avoid bias. All pregnancies, i.e. spontaneous conceptions and conceptions by fertility treatment (7% and 5% of all pregnancies in CCSs and controls, respectively) were included in the analyses. Obstetric outcomes were evaluated not only in the group of women who ever pursued pregnancy but in the total group of participating women and all pregnancies were included with the exception of twin gestations (2% and 1% of all pregnancies of CCSs and controls, respectively) and on-going pregnancies (5% and 3% of all pregnancies for CCSs and controls, respectively).

Multivariable logistic regression analyses were used to calculate odds ratios (ORs) with 95% confidence intervals (CIs) for ever having been pregnant (yes/no). These analyses were adjusted for age and educational level at time of study.

Differences in TTP and obstetric outcomes between CCSs and controls were evaluated by multivariable analyses using Generalized Estimating Equations (GEE) models to account for potential correlations between pregnancies in the same woman. TTP was evaluated by calculating ratios with 95% CIs, as a measure of the change in TTP (in months) for survivors compared to controls. Overall differences between CCSs and controls regarding TTP were analyzed for first, second, and third pregnancies separately. Since the distribution of data on TTP was highly skewed, data were log-transformed prior to analyses. For the analysis concerning adverse obstetric outcomes, each outcome (i.e. still birth, miscarriage, or induced abortion) was evaluated relative to a live birth, meaning that only the positive outcome was included in the denominator. For pregnancies ending in a live birth, the following outcomes were dichotomized for each live birth: preterm delivery, delivery of a small for gestational age infant, and delivery by caesarean section. Analyses of TTP and obstetric outcomes were adjusted for maternal age at time of pregnancy and educational level, both predictors of TTP (Mutsaerts et al. 2012), preterm delivery, and SGA offspring (Ruiz et al. 2015) in the general population.

Effects of diagnosis- and treatment-related factors on pregnancy rates, TTP, and adverse obstetric outcomes were assessed using five different multivariable models, with each model evaluating a specific factor: (1) type of diagnosis; (2) age group at diagnosis; (3) Cyclophosphamide Equivalent Dose (CED) score (Green et al. 2014); (4) radiotherapy body site; (5) having had a stem cell transplantation (SCT). In addition to these models, the effect of individual alkylating agents on pregnancy rates and TTP was evaluated. Analyses regarding the effect of radiotherapy body site on the study outcomes were additionally corrected for treatment with alkylating agents, whereas analyses concerning the effect of chemotherapy were corrected for treatment with gonadotoxic radiotherapy (total body irradiation (TBI) and/or lower abdominal/pelvic irradiation) (van den Berg et al. 2018). For all analyses, the control group was used as the reference group. Only obstetric outcomes reported in ≥ 5 CCSs were included in analyses. When results showed a specific (age group at) diagnosis to be (borderline) significantly associated with one of the outcomes, additional analyses were performed within this group (numbers permitting). For this purpose, radiotherapy body sites and chemotherapy (alkylating agents yes/no) were added to the regression model, thereby further investigating which specific types of treatment seem to underlie this association.

A *p*-value of < 0.05 (two sided) was considered statistically significant. All analyses were conducted using Statistical package for Social Sciences (SPSS) version 22.0.

### Ethical approval

Approval was obtained from the Medical Ethics Review Committees of the VU University Medical Center and written informed consent was obtained from all participants.

## Results

Of the 1749 CCSs and 1673 controls invited to participate in the study, 1095 (63%) and 812 (49%) completed the questionnaire (Table 1). Median [interquartile range (IQR)] age at time of study was 28.7 (12.5) and 32.7 (12.6) years for CCSs and controls, respectively (*p* < 0.001). Moreover, adjusted for age at study, CCSs were less likely to have a high educational level and to have ever been (living as) married at time of study (both *p* < 0.001). Survivors’ median (IQR) age at diagnosis was 6.4 (8.4) years, and the majority was diagnosed with leukaemia (36%) or lymphoma (16%).

**Table 1:** Demographic characteristics of all participating childhood cancer survivors (CCSs) and controls

	CCSs (*n* = 1095)	Controls (*n* = 812)	*p*-value
Age at time of study (years)			
Median (IQR)	28.5 (12.4)	32.7 (12.7)	< 0.001
Education^a^			< 0.001^b^
Low	93 (8.6)	24 (3.0)	
Medium	678 (62.4)	362 (44.9)	
High	315 (29.0)	420 (52.1)	
Marital status			< 0.001^b^
Never married	308 (28.3)	146 (18.0)	
Ever married/living as married	782 (71.7)	664 (82.0)	
BMI			
Median (IQR)	22.9 (5.6)	23.0 (4.8)	0.94
Age at cancer diagnosis (years)			
Median (IQR)	6.4 (8.4)	–	
< 10	739 (67.5)		
≥ 10 to < 13	164 (15.0)		
≥ 13	192 (17.5)		
Diagnosis			
Leukaemia	390 (35.6)	–	
Lymphoma	177 (16.2)	–	
CNS tumours	114 (10.4)	–	
Neuroblastoma and other peripheral nervous cell tumours	69 (6.3)	–	
Renal tumours	123 (11.2)	–	
Bone tumours	69 (6.3)	–	
Soft tissue sarcoma	75 (6.8)	–	
Germ cell tumours	46 (4.2)		
Other^c^	32 (2.9)	–	
Time since diagnosis (years)			
Median (IQR)	21.9 (12.0)	–	
CED-score^d^			
0	516 (48.5)	–	
> 0 to ≤ 4000 mg/m^2^	233 (21.9)	–	
> 4000 to ≤ 8000 mg/m^2^	142 (13.3)	–	
> 8000 mg/m^2^	174 (16.3)	–	
Radiotherapy body site			
No RT	640 (59.0)	–	
Cranial/spinal RT^e^	232 (21.4)	–	
Low dose (< 20 Gy)	48 (22.3)		
Medium dose (20–30 Gy)	86 (40.0)		
High dose (≥ 30 Gy)	81 (37.7)		
Lower abdominal/pelvic RT^f^	88 (8.0)	–	
Low dose (< 20 Gy)	36 (40.9)		
Medium dose (20–30 Gy)	20 (22.7)		
High dose (≥ 30 Gy)	32 (36.4)		
TBI^g^	33 (3.0)	–	
Other RT	92 (8.5)	–	
Stem cell transplantation			
No	1039 (94.9)	–	
Yes	46 (4.2)		

Values represent the number (%) of women, unless indicated otherwise. The subcategories may not add up to the total number of women due to missing values

*BMI* body mass index, *CED* cyclophosphamide equivalent dose, *CNS* central nervous system, *RT* radiotherapy, *TBI* total body irradiation

^a^Categorized as low: up to and including lower technical and vocational training; medium: up to and including secondary technical and vocational training; high: up to and including higher technical and vocational training and university

^b^Corrected for age at time of study

^c^Other diagnoses include thyroid carcinomas (*n* = 6), retinoblastomas (*n* = 3), hepatoblastomas (*n* = 3), nasopharyngeal carcinomas (*n* = 3), other (*n* = 3)

^d^Cyclophosphamide equivalent dose (CED) score (Green et al. 2014)

^e^Includes cranial, spinal, and craniospinal RT. 14/232 (6%) patients treated with cranial/spinal RT also received other RT

^f^4/88 (5%) patients treated with lower abdominal/pelvic RT also received cranial/spinal RT and 12/88 (14%) patients also received other RT

^g^3/33 (9%) patients treated with TBI also received cranial/spinal RT

### Pregnancy rates

In total, 482 out of 1095 (44%) survivors and 422 out of 812 (52%) controls ever pursued a pregnancy. Among this subgroup, 393 survivors (82%) and 383 (91%) controls reported at least one pregnancy at time of study (OR 0.5, 95%CI 0.4–0.8). The mean [standard deviation (SD)] maternal age at first pregnancy was 27.4 (4.2) and 28.6 (4.7) years for CCSs and controls, respectively (*p* = 0.05). After adjustment for educational level, results showed a trend towards a lower maternal age at first pregnancy for CCSs compared to controls (*p* = 0.08). Survivors of CNS or renal tumors, as well as those treated with high-dose cranial/spinal radiotherapy, medium and high-dose abdominal/pelvic radiotherapy, TBI, or stem cell transplantation, were significantly less likely to have ever been pregnant (Supplementary Table 1). None of the CED-score categories, nor any of the individual alkylating agents (yes/no), were significantly association with ever having been pregnant (data not shown).

### Time to pregnancy

Among those ever pregnant in the group of women who ever pursue a pregnancy, data on TTP were available for 721 and 787 pregnancies from 378 CCSs and 369 controls, respectively. Overall TTP did not appear significantly longer in CCSs compared to controls [1.1 times longer (*p* = 0.09)]. However, in survivors of CNS and renal tumours TTP was significantly longer (Table 2), in renal tumours particularly following treatment with lower abdominal/pelvic radiotherapy (ratio 1.5, 95%CI 1.0–2.2). For CNS survivors, no particular treatment factor was associated with a longer TTP (data not shown). None of the CED-score categories, nor any of the individual alkylating agents (yes/no) was significantly associated with TTP (data not shown). Only lower abdominal/pelvic radiotherapy was borderline significantly associated with a longer TTP (*p* = 0.08).

**Table 2:** Diagnosis- and treatment-related factors associated with time to pregnancy according to five different models: (1) type of diagnosis; (2) age at diagnosis; (3) CED score; (4) radiotherapy site; (5) stem cell transplantation

	Total number of pregnancies	Time to pregnancy (months); Median (IQR)	Ratio (95% CI)
Controls	787	3.0 (1.0–7.5)	Ref.
Survivors	721	3.0 (1.0–8.0)	1.1 (1.0–1.2)^a^
*Model 1: type of diagnosis*^a^			
Controls	787	3.0 (1.0–7.5)	Ref.
Leukaemia	236	3.0 (1.0–7.3)	1.1 (0.9–1.2)
Lymphoma	164	3.0 (1.0–8.0)	1.1 (0.9–1.4)
CNS tumours	27	7.0 (3.0–12.0)	2.0 (1.3–3.0)
Neuroblastoma and other peripheral nervous cell tumours	60	3.5 (1.3–9.0)	1.0 (0.8–1.3)
Renal tumours	66	3.5 (1.4–12.0)	1.3 (1.0–1.8)
Bone tumours	72	3.0 (2.0–5.8)	1.0 (0.8–1.3)
Soft tissue sarcoma	49	3.0 (1.8–6.0)	0.9 (0.7–1.2)
Germ cell tumours	24	2.0 (1.0–3.8)	0.9 (0.5–1.5)
Other	23	4.0 (2.0–9.0)	1.3 (0.8–1.9)
*Model 2: age at diagnosis (years)*^a^			
Controls	787	3.0 (1.0–7.5)	Ref.
< 10	408	3.0 (1.5–9.0)	1.1 (1.0–1.3)
≥ 10 to < 13	120	3.0 (1.0–8.8)	1.1 (0.9–1.4)
≥ 13	193	3.0 (1.0–7.5)	1.1 (0.9–1.3)
*Model 3: CED score*^b^			
Controls	787	3.0 (1.0–7.5)	Ref.
0	354	3.0 (1.0–9.0)	1.1 (0.9–1.2)
> 0 to ≤ 4000 mg/m^2^	100	3.0 (1.0–7.0)	1.0 (0.8–1.3)
> 4000 to ≤ 8000 mg/m^2^	104	4.0 (1.3–8.0)	1.1 (0.9–1.4)
> 8000 mg/m^2^	142	3.0 (2.0–8.0)	1.1 (0.9–1.4)
*Model 4: radiotherapy body site*^c^			
Controls	787	3.0 (1.0–7.5)	Ref.
No RT	377	3.0 (1.0–7.0)	1.0 (0.9–1.2)
Cranial/spinal RT	161	4.0 (1.0–9.0)	1.2 (0.9–1.5)
Low dose (< 20 Gy)	47	3.0 (1.0–7.0)	1.2 (0.7–2.0)
Medium dose (20–30 Gy)	73	4.0 (2.0–12.0)	1.3 (0.9–1.8)
High dose (≥ 30 Gy)	38	6.0 (1.0–9.8)	1.3 (0.8–2.1)
Lower abdominal/pelvic RT	64	4.8 (2.0–10.5)	1.3 (1.0–1.8)
Low dose (< 20 Gy)	26	6.5 (2.0–14.5)	1.7 (0.9–2.9)
Medium dose (20–30 Gy)	15	3.0 (0.0–6.0)	1.0 (0.6–1.9)
High dose (≥ 30 Gy)	23	5.0 (3.0–12.0)	1.5 (1.0–2.4)
TBI	7	5.0 (4.0–10.0)	1.2 (0.5–2.7)
Other RT	104	3.0 (2.0–6.0)	1.0 (0.8–1.3)
*Model 5: stem cell transplantation*^a^			
Controls	787	3.0 (1.0–7.5)	Ref.
No	696	3.0 (1.0–8.0)	1.1 (1.0–1.2)
Yes	14	4.8 (0.8–10.5)	1.2 (0.6–2.3)

*CED* cyclophosphamide equivalent dose, *CNS* central nervous system, *RT* radiotherapy, *TBI* total body irradiation

^a^Corrected for age at time of pregnancy and educational level

^b^Corrected for age at time of pregnancy, educational level, and gonadotoxic radiotherapy (i.e. lower abdominal/pelvic RT and/or TBI) (yes or no)

^c^Corrected for age at time of pregnancy, educational level and alkylating agent therapy (yes or no)

TTP analyzed for first, second, and third pregnancies separately did not differ significantly between CCSs and controls nor did TTP for pregnancies by spontaneous conception and by conception using assisted reproductive techniques (Supplementary Table 2).

### Obstetric outcomes

Among all women who participated in the study, 415 survivors and 401 controls reported a total of 845 and 890 pregnancies, respectively. Data on outcomes of 786 (93%) and 853 (96%) pregnancies among 392 survivors and 387 controls, respectively, were available for analyses. Overall, no differences were found between CCSs and controls regarding the probability of experiencing a still birth, miscarriage, or induced abortion (OR 2.0, 95%CI 0.5–7.6; OR 1.2, 95%CI 0.8–1.6; OR 0.7, 95%CI 0.5–1.2, respectively) (Table 3). However, a significantly increased probability of miscarriage was found specifically among survivors of leukemia (OR 1.6, 95%CI 1.1–2.3) which seemed to be related to treatment with TBI, administered as part of conditioning regimen for SCT, although numbers were too small to perform valid statistical tests. Of the 11 pregnancies among leukaemia survivors who received TBI, eight (73%) ended in a miscarriage and one (9%) in a still birth. For the renal tumour survivor group as a whole the probability of miscarriage was also increased, reaching borderline significance (OR 1.9, 95%CI 1.0–3.5). Additional analysis showed particularly renal tumour survivors treated with lower abdominal/pelvic radiotherapy to be at increased risk (OR 2.4, 95%CI 1.1–5.1). In addition, women who received a SCT had a significantly increased risk of a miscarriage (OR 8.2, 95%CI 2.2–30.0). None of the investigated diagnosis- or treatment-related factors appeared to be associated with in an increased risk of induced abortion.

**Table 3:** Diagnosis- and treatment-related factors associated with miscarriage, induced abortion and still birth, according to five different models: (1) type of diagnosis; (2) age at diagnosis; (3) CED score; (4) radiotherapy site; (5) stem cell transplantation

	Total number of pregnancies	Pregnancy outcome					
		Live birth	Miscarriage	Induced abortion	Still birth	Miscarriage OR (95% CI)	Induced abortion OR (95% CI)
Controls	853	651 (76.3)	138 (16.2)	60 (7.0)	4 (0.5)	Ref.	Ref.
Survivors	786	592 (75.3)	138 (17.6)	48 (6.1)	8 (1.0)	1.2 (0.8–1.6)^a^	0.7 (0.5–1.2)^a^
*Model 1: Type of diagnosis*^a^							
Controls	853	651 (76.3)	138 (16.2)	60 (7.0)	4 (0.5)	Ref.	Ref.
Leukaemia	269	191 (71.0)	57 (21.2)	18 (6.7)	3 (1.1)	1.6 (1.1–2.3)	1.0 (0.5–2.0)
Lymphoma	166	134 (80.7)	19 (11.4)	12 (7.2)	1 (0.6)	0.8 (0.4–1.3)	0.6 (0.2–1.5)
CNS tumours	33	25 (75.8)	6 (18.2)	2 (6.1)	0	1.3 (0.5–3.4)	–
Neuroblastoma and other peripheral nervous cell tumours	56	45 (80.4)	7 (12.5)	1 (1.8)	3 (5.4)	0.7 (0.3–2.1)	–
Renal tumours	72	53 (73.6)	17 (23.6)	2 (2.8)	0	1.9 (1.0–3.5)	–
Bone tumours	76	61 (80.3)	10 (13.2)	4 (5.3)	1 (1.3)	0.5 (0.2–1.6)	–
Soft tissue sarcoma	55	43 (78.2)	9 (16.4)	3 (5.5)	0	1.2 (0.6–2.5)	–
Germ cell tumours	31	21 (67.7)	8 (25.8)	2 (6.5)	0	1.5 (0.5–4.4)	–
Other	28	19 (67.9)	5 (17.9)	4 (14.3)	0	0.6 (0.2–2.0)	–
*Model 2: Age at diagnosis (years)*^a^							
Controls	853	651 (76.3)	138 (16.2)	60 (7.0)	4 (0.5)	Ref.	Ref.
< 10	442	332 (75.1)	75 (17.0)	30 (6.8)	5 (1.1)	1.1 (0.8–1.6)	0.8 (0.5–1.5)
≥ 10 to < 13	139	105 (75.5)	23 (16.5)	8 (5.8)	3 (2.2)	1.2 (0.7–2.0)	0.7 (0.3–1.8)
≥ 13	205	155 (75.6)	40 (19.5)	10 (4.9)	0	1.2 (0.8–1.9)	0.5 (0.2–1.4)
*Model 3: CED score*^b^							
Controls	853	651 (76.3)	138 (16.2)	60 (7.0)	4 (0.5)	Ref.	Ref.
0	394	301 (76.4)	68 (17.3)	23 (5.8)	2 (0.5)	1.1 (0.8–1.6)	0.9 (0.5–1.7)
> 0 to ≤ 4000 mg/m^2^	121	78 (64.5)	33 (27.3)	9 (7.4)	1 (0.8)	1.4 (0.8–2.5)	0.6 (0.2–1.9)
> 4000 to ≤ 8000 mg/m^2^	102	82 (80.4)	11 (10.8)	6 (5.9)	3 (2.9)	0.5 (0.2–1.3)	1.0 (0.4–2.5)
> 8000 mg/m^2^	146	114 (78.1)	21 (14.4)	9 (6.2)	2 (1.4)	0.8 (0.5–1.4)	0.7 (0.2–2.0)
*Model 4: Radiotherapy body site*^c^			chi				
Controls	853	651 (76.3)	138 (16.2)	60 (7.0)	4 (0.5)	Ref.	Ref.
No RT	400	305 (76.3)	61 (15.3)	30 (7.5)	4 (1.0)	1.1 (0.7–1.7)	0.9 (0.5–1.9)
Cranial/spinal RT	183	141 (77.0)	35 (19.1)	5 (2.7)	2 (1.1)	1.3 (0.8–2.2)	0.8 (0.3–2.1)
Lower abdominal/pelvic RT	72	54 (75.0)	17 (23.6)	1 (1.4)	0	1.8 (0.9–3.5)	–
TBI	11	2 (18.2)	8 (72.7)	0	1 (9.1)	–	–
Other RT	113	86 (76.1)	15 (13.3)	11 (9.7)	1 (0.9)	1.1 (0.6–2.1)	1.1 (0.4–3.1)
*Model 5: Stem cell transplantation*^a^							
Controls	853	651 (76.3)	138 (16.2)	60 (7.0)	4 (0.5)	Ref.	Ref.
No	756	577 (76.3)	125 (16.5)	47 (6.2)	7 (0.9)	1.1 (0.8–1.4)	0.7 (0.4–1.2)
Yes	16	6 (37.5)	9 (56.3)	0	1 (6.3)	8.2 (2.2–30.0)	–

Values represent the number (%) of women, unless indicated otherwise. The subcategories may not add up to the total number of women due to missing values

*CED* cyclophosphamide equivalent dose, *CNS* central nervous system, *RT* radiotherapy, *TBI* total body irradiation

^a^Corrected for age at time of pregnancy and educational level

^b^Corrected for age at time of pregnancy, educational level, and gonadotoxic radiotherapy (i.e. lower abdominal/pelvic RT and/or TBI) (yes or no)

^c^Corrected for age at time of pregnancy, educational level, and alkylating agent therapy (yes or no)

Overall, 57 (10%) and 31 (5%) live births from CCSs and controls, respectively, were delivered preterm (OR 2.0, 95%CI 1.2–3.5) (Table 4). This risk was significantly higher specifically among survivors of renal tumours, bone tumours, and soft tissue sarcomas. The risk of a preterm delivery among renal tumour survivors was independent of having received lower abdominal/pelvic radiotherapy (data not shown). For survivors of bone tumours and soft tissue sarcomas, the numbers were too small to further investigate which type of treatment primarily contributed to a preterm delivery. Moreover, CCSs diagnosed before the age of 10 years had a significantly higher probability of a preterm delivery, which seemed to be related to treatment with treatment with lower abdominal/pelvic radiotherapy. Overall, treatment with lower abdominal/pelvic radiotherapy or cranial/spinal radiotherapy, but also with CED-score > 4000–8000, resulted in a higher risk of a preterm delivery.

**Table 4:** Diagnosis- and treatment-related factors associated with obstetric outcomes according to five different models: (1) type of diagnosis; (2) age at diagnosis; (3) CED score; (4) radiotherapy site; (5) stem cell transplantation

	Total number of live births	Preterm delivery		SGA		Caesarean section	
		*N* (%)^a^	OR (95% CI)	*N* (%)^b^	OR (95% CI)	*N* (%)^c^	OR (95% CI)
Controls	651	31 (4.8)	Ref.	61 (9.8)	Ref.	77 (12.3)	Ref.
Survivors	592	57 (9.8)	2.0 (1.2–3.5)^d^	59 (10.7)	1.1 (0.7–1.7)^d^	108 (19.2)	1.8 (1.2–2.6)^d^
*Model 1: Type of diagnosis*^d^							
Controls	651	31 (4.8)	Ref.	61 (9.8)	Ref.	77 (12.3)	Ref.
Leukaemia	191	13 (7.0)	1.4 (0.6–2.9)	16 (9.0)	0.8 (0.4–1.5)	35 (19.3)	1.8 (1.1–3.0)
Lymphoma	134	12 (9.2)	1.7 (0.7–3.7)	11 (8.7)	0.8 (0.4–1.7)	16 (12.6)	0.9 (0.5–1.9)
CNS tumours	25	4 (16.0)	–	4 (17.4)	–	6 (24.0)	2.2 (0.7–4.6)
Neuroblastoma and other peripheral nervous cell tumours	45	2 (4.4)	–	8 (19.0)	2.4 (1.1–5.3)	8 (18.6)	1.9 (0.8–4.6)
Renal tumours	53	10 (18.9)	4.9 (2.1–11.6)	3 (6.0)	–	12 (24.0)	2.7 (1.2–6.1)
Bone tumours	61	7 (11.9)	2.8 (1.1–7.0)	8 (14.0)	1.7 (0.7–4.0)	14 (24.1)	2.3 (1.1–5.1)
Soft tissue sarcoma	43	7 (16.3)	4.0 (1.3–12.6)	7 (17.5)	2.0 (0.7–5.8)	13 (31.7)	3.4 (1.4–8.4)
Germ cell tumours	21	1 (4.8)	–	1 (4.8)	–	2 (9.5)	–
Other	19	1 (5.3)	–	1 (5.3)	–	2 (10.5)	–
*Model 2: Age at diagnosis (years)*^d^							
Controls	651	31 (4.8)	Ref.	61 (9.8)	Ref.	77 (12.3)	Ref.
< 10	332	38 (11.7)	2.5 (1.4–4.4)	31 (10.0)	1.0 (0.6–1.6)	68 (21.4)	2.1 (1.4–3.2)
≥ 10 to< 13	105	9 (8.7)	1.9 (0.8–4.5)	12 (12.0)	1.2 (0.6–2.6)	15 (15.3)	1.4 (0.7–2.9)
≥ 13	155	10 (6.7)	1.3 (0.5–3.4)	16 (11.5)	1.2 (0.6–2.5)	25 (17.1)	1.4 (0.8–2.6)
*Model 3: CED score*^e^							
Controls	651	31 (4.8)	Ref.	61 (9.8)	Ref.	77 (12.3)	Ref.
0	301	28 (9.6)	1.9 (1.0–3.6)	23 (8.3)	0.8 (0.5–1.4)	57 (19.5)	2.0 (1.2–3.1)
> 0 to ≤ 4000 mg/m^2^	78	6 (7.7)	1.1 (0.4–3.1)	11 (15.1)	1.5 (0.6–3.9)	15 (21.4)	2.1 (1.0–4.4)
> 4000 to ≤ 8000 mg/m^2^	82	12 (14.8)	3.0 (1.3–7.4)	9 (11.4)	1.2 (0.5–2.6)	24 (30.0)	2.9 (1.5–5.6)
> 8000 mg/m^2^	114	10 (8.8)	1.6 (0.7–3.6)	14 (13.0)	1.4 (0.7–2.8)	12 (11.5)	0.8 (0.4–1.8)
*Model 4: Radiotherapy body site*^f^							
Controls	651	31 (4.8)	Ref.	61 (9.8)	Ref.	77 (12.3)	Ref.
No RT	305	28 (9.4)	2.1 (1.0–4.3)	19 (6.6)	0.5 (0.3–1.0)	45 (15.8)	1.6 (0.9–2.7)
Cranial/spinal RT	141	14 (10.1)	2.1 (1.0–4.5)	19 (15.0)	1.2 (0.6–2.3)	36 (26.1)	2.8 (1.5–5.0)
Lower abdominal/pelvic RT	54	8 (14.8)	4.0 (1.6–10.1)	4 (8.3)	–	11 (21.2)	2.1 (0.9–5.2)
TBI	2	1 (50.0)	–	1 (50.0)	–	0	–
Other RT	86	6 (7.2)	1.6 (0.6–4.6)	15 (18.8)	1.5 (0.6–3.7)	15 (18.3)	1.9 (0.9–4.2)
*Model 5: Stem cell transplantation*							
Controls	651	31 (4.8)	Ref.	61 (9.8)	Ref.	77 (12.3)	Ref.
No	577	56 (9.9)	2.1 (1.2–3.6)	57 (10.6)	1.1 (0.7–1.7)	106 (19.3)	1.8 (1.2–2.6)
Yes	6	0	–	0	–	1 (16.7)	–

Values represent the number (%) of women, unless indicated otherwise. The subcategories may not add up to the total number of women due to missing values

*CED* cyclophosphamide equivalent dose, *CNS* central nervous system, *RT* radiotherapy, *SGA* small for gestational age, *TBI* total body irradiation

^a^Percentage of the live births for whom the gestational week was known

^b^Percentage of the live births for whom SGA could be calculated

^c^Percentage of the live births for whom the method of delivery was known

^d^Corrected for age at time of pregnancy and educational level

^e^Corrected for age at time of pregnancy, educational level, and gonadotoxic radiotherapy (i.e. lower abdominal/pelvic RT and/or TBI) (yes or no)

^f^Corrected for age at time of pregnancy, educational level, and alkylating agent therapy (yes or no)

No significant differences were found regarding the risk of SGA offspring among CCSs (11%) compared to controls (10%), with the exception of neuroblastoma survivors (OR 2.4, 95%CI 1.1–5.3) in whom no specific type of treatment associated with this finding could be identified.

Furthermore, 108 (19%) and 77 (12%) of live births in CCSs and controls, respectively, were delivered via caesarean section (OR 1.8, 95%CI 1.2–2.6). Survivors of leukaemia, renal tumours, bone tumours, and soft tissue sarcoma were at a significantly increased risk of a caesarean section (Table 4). Within the group of leukaemia survivors, treatment with cranial/spinal radiotherapy was significantly associated with ever having had a caesarean section (OR 3.0, 95%CI 1.4–6.1), while lower abdominal/pelvic radiotherapy appeared to be the main associated factor within the group of renal tumour survivors (OR 2.7, 95%CI 1.0–7.4). All soft tissue sarcoma survivors who received cranial/spinal radiotherapy delivered via caesarean section, whereas for bone tumour survivors no specific types of chemo- or radiotherapy were associated with delivering via caesarean section. Moreover, survivors diagnosed before the age of 10 years were significantly more likely to have delivered via a caesarean section, which was related to treatment with cranial/spinal radiotherapy. Moreover, overall analyses showed that treatment with cranial/spinal radiotherapy resulted in a significantly higher probability of delivering via caesarean section (OR 2.8, 95%CI 1.5–5.0).

## Discussion

This is the first study which assessed pregnancy rates among female CCSs and controls who ever pursued pregnancy. Moreover, this is one of the first studies assessing specific diagnosis- and treatment-related factors associated with pregnancy rates, TTP and adverse obstetric outcomes using a large, nationwide, cohort of female CCSs.

Our results show that the chance of becoming pregnant was significantly lower for CCSs compared to controls, which is in line with previous studies (Green et al. 2009b; Chow et al. 2016; Armuand et al. 2017). In most of these studies results regarding pregnancy rates were adjusted for marital status, as an alternative way to correct for the fact that not all study participants did actually want to become pregnant. These studies appeared to provide similar results as our study, which is reassuring in terms of the robustness of our findings. However, the fact that our analyses were performed among the subgroup of participants who ever pursued pregnancy, has yielded more valid estimates regarding the actual chance of becoming pregnant following childhood cancer. Similar to Green et al., high dose cranial radiotherapy (> 30 Gy) and medium and high dose abdominal/pelvic radiotherapy were found as risk factors for reduced pregnancy rates in addition to TBI or SCT. Conditioning treatment for SCT often includes chemotherapeutic agents such as busulfan, lomustine or cyclophosphamide, all of which have been associated with reduced pregnancy rates (Green et al. 2009b; Chow et al. 2016). Due to the limited numbers of patients who received these alkylating agents in our study, we were unable to identify this association.

Overall, no difference in TTP between CCSs and controls was found. This contrasts to Barton et al. who reported a significantly increased TTP for CCSs (Barton et al. 2013). However, as opposed to this study, we included all pregnancies and corrected our analyses for maternal age at pregnancy and maternal educational level at time of study, both factors known to be associated with TTP (Mutsaerts et al. 2012). Our results may indicate that CCSs are more aware of the possible impact of their former treatment on reproductive function, as a result of improved and more frequent counseling. Therefore, they may start attempting to become pregnant at a younger age, thereby counteracting the adverse impact of treatment on TTP. This seems to be substantiated by our finding that CCSs were younger than controls at the time of their first pregnancy. After adjustment for educational level, a trend towards a younger age at first pregnancy among CCSs was still observed. Moreover, TTP was investigated not only for first pregnancies but for all pregnancies. Our results revealed no differences in TTP between CCSs and controls for any of the subsequent pregnancies. These findings suggest that although ovarian reserve may be decreased in female CCSs (van den Berg et al. 2018), the quality of the oocytes in the remaining follicle pool is not compromised (Somigliana et al. 2018) as opposed to ageing women where both the quantity and quality of the remaining follicles are reduced (Bruin et al. 2004; Wallace and Kelsey 2010).

Survivors of CNS and renal tumours had a significantly longer TTP compared to controls, particularly after lower abdominal/pelvic radiotherapy in renal tumour survivors which may have resulted in a decreased ovarian reserve or a damaged uterus not facilitating conception and/or implantation (Green et al. 2009a; van den Berg et al. 2018; Teh et al. 2014). For CNS-tumour survivors, none of the radiotherapy body sites significantly contributed to a longer TTP suggesting that having had a CNS tumour, rather than treatment with radiotherapy is associated with longer TTP. It should however be noted that the number of pregnancies within these subgroups was low. Brinkman et al. reported that survivors of childhood CNS tumours, treated with and without cranial radiotherapy, are at an increased risk of neurocognitive impairment (Brinkman et al. 2016). As a consequence, they may have fertility-related knowledge deficits and incorrect perceptions regarding their reproductive window leading to more problems when trying to conceive (Hammarberg et al. 2013). However, TTP was less than 12 months for the majority of the CNS (78%) and renal tumor survivors (79%), indicating that clinically they are not considered subfertile (Practice Committee of American Society for Reproductive M 2008).

It has been suggested previously that cranial irradiation increases the risk of miscarriage, possibly through impairment of the hypothalamic-pituitary-ovarian-axis function (Green et al. 2002; Bath et al. 2001). However, this was not confirmed in our study, nor in the study by Reulen et al. (2009). Further research is needed to investigate the exact effect of cranial radiotherapy and associated pituitary hormone deficits on the risk of miscarriages. In addition, although we found SCT to be associated with an increased risk of miscarriage, numbers were too small to further investigate which factor primarily contributed to this increased risk, i.e. TBI or other aspects of SCT. However, as in another study (Sanders et al., 1996), our results suggest a role for TBI since 8/11 (73%) pregnancies of survivors treated with TBI as part of a SCT resulted in a miscarriage, whereas only 1/6 pregnancies (17%) of survivors not treated with TBI prior to SCT resulted in a miscarriage.

In line with previous studies, survivors were at increased risk of a preterm delivery in general (Madanat-Harjuoja et al. 2010; Mueller et al. 2009) and in particular survivors who received lower abdominal/pelvic radiotherapy (Reulen et al. 2009; 2017; Lie Fong et al. 2010; Green et al. 2010). Radiotherapy to the uterus during childhood has previously been associated with damage to the uterus vasculature, its elasticity, and volume (Larsen et al. 2003; Wo and Viswanathan 2009; Loo et al. 2019), restricting a survivors ability to carry the fetus to term. However, since in our study the risk of preterm delivery appeared independent of having received lower abdominal/pelvic radiotherapy, other factors than cancer treatment may also contribute, such as hypertension or diabetes mellitus (as a result of either the pregnancy itself or previous cancer treatment). In our population 27% (14/52) of the survivors who ever delivered prematurely, also suffered from hypertension during pregnancy, while this proportion was 12% (3/25) among controls. No diabetes during pregnancy was reported. In addition, a significantly higher risk of a preterm delivery was found in CCSs treated with lower abdominal/pelvic radiotherapy before the age of 10 years. This is in line with previous studies indicating that radiotherapy to the uterus administered pre-pubertally is associated with a smaller uterus in adulthood, as uterine development is not completed before the onset of puberty (Bath et al. 1999; Bruin et al. 2009), thereby increasing the risk of delivering preterm. Finally, we found soft tissue sarcoma survivors to be at increased risk of preterm delivery. Although actual numbers withheld us from performing additional analyses into the specific type of treatment responsible for this increased risk, the location of the tumour seems critical: among survivors whose sarcoma was located in the uterus, four of the five pregnancies (80%) resulted in a preterm delivery, whereas this proportion was 8% (3/38 pregnancies) among those whose primary sarcoma originated from non-uterine soft tissues.

Our study showed that survivors had a higher risk of delivering via caesarean section compared to controls, which is in line with previous studies. (Reulen et al. 2017; van der Kooi et al. 2018; Melin et al. 2015). Possibly a history of cancer may lead to increased surveillance and a lower threshold for interventions, such as caesarean sections.

Strengths of the current study, besides the fact that it is the first study evaluating TTP in all pregnancies, include the evaluation of the chance of becoming pregnant among the group that actually pursued pregnancy, the inclusion of a large control group, and the availability of detailed cancer treatment information. Moreover, we evaluated the risk of each adverse pregnancy outcome relative to a live birth, while previous studies evaluated each adverse pregnancy outcome relative to all other pregnancy outcomes, thereby underestimating the actual risks.

Our study also has some limitations. First, our study may have been subject to participation bias, since participating CCSs may differ from non-participating CCSs. However, participating and non-participating CCSs did not differ regarding age at study, age at diagnosis, and type of diagnosis (Supplementary Table 3). The proportion of CCSs treated with chemotherapy only was significantly higher among participants compared to non-participants. This may indicate that the more heavily treated CCSs did not participate, which would imply that the reported risks of adverse obstetric outcomes may be an underestimation of the actual risk. Furthermore, although it is known that TTP and obstetric outcomes are associated with factors such as BMI, smoking behavior, and menstrual cycle length, our analyses were not corrected for these factors, since for these factors only data at time of study were available and not at time of pregnancy, which may have occurred years earlier. Finally, women who tried but failed to conceive were not included in the analyses regarding TTP. Consequently, our findings on TTP are valid for CCSs who have proven to be fertile only.

In conclusion, although the chance of becoming pregnant is significantly lower for CCSs compared to controls, it is reassuring that overall, CCSs who become pregnant are not at increased risk of a longer TTP, stillbirth, miscarriage, or induced abortion. Nevertheless, the risk of delivering preterm or via caesarean section is higher among CCSs compared to controls and specific subgroups of CCSs seem to be at increased risk of longer TTP and several adverse obstetric outcomes, especially those who received lower abdominal/pelvic irradiation. Close obstetric monitoring for these survivors is of high importance. Moreover, our results can be incorporated in clinical guidelines regarding obstetric care in female CCSs.

## Electronic supplementary material

Below is the link to the electronic supplementary material.

Supplementary file1 (DOCX 25 kb)

## References

[CR1] Anderson RA, Brewster DH, Wood R, Nowell S, Fischbacher C, Kelsey TW (2018). The impact of cancer on subsequent chance of pregnancy: a population-based analysis. Hum Reprod.

[CR2] Armuand G, Skoog-Svanberg A, Bladh M, Sydsjo G (2017). Reproductive patterns among childhood and adolescent cancer survivors in Sweden: a population-based matched-cohort study. J Clin Oncol.

[CR3] Barton SE, Najita JS, Ginsburg ES, Leisenring WM, Stovall M, Weathers RE (2013). Infertility, infertility treatment, and achievement of pregnancy in female survivors of childhood cancer: a report from the Childhood Cancer Survivor Study cohort. Lancet Oncol.

[CR4] Bath LE, Critchley HO, Chambers SE, Anderson RA, Kelnar CJ, Wallace WH (1999). Ovarian and uterine characteristics after total body irradiation in childhood and adolescence: response to sex steroid replacement. Br J Obstet Gynaecol.

[CR5] Bath LE, Anderson RA, Critchley HOD, Kelnar CJH, Wallace WH (2001). Hypothalamic-pituitary-ovarian dysfunction after prepubertal chemotherapy and cranial irradiation for acute leukeamia. Hum Reprod.

[CR10] Brinkman TM, Krasin MJ, Liu W, Armstrong GT, Ojha RP, Sadighi ZS (2016). Long-term neurocognitive functioning and social attainment in adult survivors of pediatric CNS tumors: results from the St Jude Lifetime Cohort Study. J Clin Oncol.

[CR12] Chemaitilly W, Mertens AC, Mitby P, Whitton J, Stovall M, Yasui Y (2006). Acute ovarian failure in the childhood cancer survivor study. J Clin Endocrinol Metab.

[CR13] Chiarelli AM, Marrett LD, Darlington GA (2000). Pregnancy outcomes in females after treatment for childhood cancer. Epidemiology.

[CR14] Chow EJ, Stratton KL, Leisenring WM, Oeffinger KC, Sklar CA, Donaldson SS (2016). Pregnancy after chemotherapy in male and female survivors of childhood cancer treated between 1970 and 1999: a report from the Childhood Cancer Survivor Study cohort. Lancet Oncol.

[CR8] de Boer EJ, den Tonkelaar I, Burger CW, van Leeuwen FE, Group OP (2005). Validity of self-reported causes of subfertility. Am J Epidemiol.

[CR9] de Bruin JP, Dorland M, Spek ER, Posthuma G, van Haaften M, Looman CW (2004). Age-related changes in the ultrastructure of the resting follicle pool in human ovaries. Biol Reprod.

[CR11] De Bruin ML, van Dulmen-den BE, van den Berg MH, Lambalk CB (2009). Fertility in female childhood cancer survivors. Endocr Dev.

[CR15] Gatta G, Botta L, Rossi S, Aareleid T, Bielska-Lasota M, Clavel J (2014). Childhood cancer survival in Europe 1999–2007: results of EUROCARE-5—a population-based study. Lancet Oncol.

[CR16] Green DM, Whitton JA, Stovall M, Mertens AC, Donaldson SS, Ruymann FB (2002). Pregnancy outcome of female survivors of childhood cancer: a report from the childhood cancer survivor study. Am J Obstet Gynecol.

[CR17] Green DM, Sklar CA, Boice JD, Mulvihill JJ, Whitton JA, Stovall M (2009). Ovarian failure and reproductive outcomes after childhood cancer treatment: results from the Childhood Cancer Survivor Study. J Clin Oncol.

[CR18] Green DM, Kawashima T, Stovall M, Leisenring W, Sklar CA, Mertens AC (2009). Fertility of female survivors of childhood cancer: a report from the childhood cancer survivor study. J Clin Oncol.

[CR19] Green DM, Lange JM, Peabody EM, Grigorieva NN, Peterson SM, Kalapurakal JA (2010). Pregnancy outcome after treatment for Wilms tumor: a report from the national Wilms tumor long-term follow-up study. J Clin Oncol.

[CR20] Green DM, Nolan VG, Goodman PJ, Whitton JA, Srivastava D, Leisenring WM (2014). The cyclophosphamide equivalent dose as an approach for quantifying alkylating agent exposure: a report from the Childhood Cancer Survivor Study. Pediatr Blood Cancer.

[CR21] Hammarberg K, Setter T, Norman RJ, Holden CA, Michelmore J, Johnson L (2013). Knowledge about factors that influence fertility among Australians of reproductive age: a population-based survey. Fertil Steril.

[CR23] Larsen EC, Muller J, Schmiegelow K, Rechnitzer C, Andersen AN (2003). Reduced ovarian function in long-term survivors of radiation- and chemotherapy-treated childhood cancer. J Clin Endocrinol Metab.

[CR24] Lie Fong S, van den Heuvel-Eibrink MM, Eijkemans MJ, Schipper I, Hukkelhoven CW, Laven JS (2010). Pregnancy outcome in female childhood cancer survivors. Hum Reprod.

[CR26] Madanat LM, Malila N, Dyba T, Hakulinen T, Sankila R, Boice JD (2008). Probability of parenthood after early onset cancer: a population-based study. Int J Cancer.

[CR27] Madanat-Harjuoja LM, Malila N, Lahteenmaki PM, Boice JD, Gissler M, Dyba T (2010). Preterm delivery among female survivors of childhood, adolescent and young adulthood cancer. Int J Cancer.

[CR28] Magelssen H, Melve KK, Skjaerven R, Fossa SD (2008). Parenthood probability and pregnancy outcome in patients with a cancer diagnosis during adolescence and young adulthood. Hum Reprod.

[CR29] Melin J, Heinavaara S, Malila N, Tiitinen A, Gissler M, Madanat-Harjuoja L (2015). Adverse obstetric outcomes among early-onset cancer survivors in Finland. Obstet Gynecol.

[CR30] Mueller BA, Chow EJ, Kamineni A, Daling JR, Fraser A, Wiggins CL (2009). Pregnancy outcomes in female childhood and adolescent cancer survivors: a linked cancer-birth registry analysis. Arch Pediatr Adolesc Med.

[CR31] Mutsaerts MA, Groen H, Huiting HG, Kuchenbecker WK, Sauer PJ, Land JA (2012). The influence of maternal and paternal factors on time to pregnancy—a Dutch population-based birth-cohort study: the GECKO Drenthe study. Hum Reprod.

[CR32] Overbeek A, van den Berg MH, Kremer LCM, van den Heuvel-Eibrink MM, Tissing WJE, Loonen JJ (2012). A nationwide study on reproductive function, ovarian reserve and risk of premature menopause in female survivors of childhood cancer: design and methodological challenges. BMC Cancer.

[CR33] Practice Committee of American Society for Reproductive M (2008). Definitions of infertility and recurrent pregnancy loss. Fertil Steril.

[CR34] Reulen RC, Zeegers MP, Wallace WH, Frobisher C, Taylor AJ, Lancashire ER (2009). Pregnancy outcomes among adult survivors of childhood cancer in the British Childhood Cancer Survivor Study. Cancer Epidemiol Biomarkers Prev.

[CR35] Reulen RC, Bright CJ, Winter DL, Fidler MM, Wong K, Guha J et al (2017) Pregnancy and labor complications in female survivors of childhood cancer: The British Childhood Cancer Survivor Study. J Natl Cancer Inst 109 10.1093/jnci/djx056 PMC5409032 28419299

[CR36] Ruiz M, Goldblatt P, Morrison J, Kukla L, Svancara J, Riitta-Jarvelin M (2015). Mother's education and the risk of preterm and small for gestational age birth: a DRIVERS meta-analysis of 12 European cohorts. J Epidemiol Community Health.

[CR37] Sanders JE, Hawley J, Levy W, Gooley T, Buckner CD, Deeg HJ (1996). Pregnancies following high-dose cyclophosphamide with or without high-dose busulfan or total-body irradiation and bone marrow transplantation. Blood.

[CR38] Schover LR (1999). Psychosocial aspects of infertility and decisions about reproduction in young cancer survivors a review. Med Pediatr Oncol.

[CR39] Schover LR, Rybickl LA, Martin BA, Bringelsen KA (1999). Having children after cancer. A pilot survey of survivors' attitudes and experiences. Cancer.

[CR40] Signorello LB, Cohen SS, Bosetti C, Stovall M, Kasper CE, Weathers RE (2006). Female survivors of childhood cancer: preterm birth and low birth weight among their children. J Natl Cancer Inst.

[CR41] Sklar CA, Mertens AC, Mitby P, Whitton J, Stovall M, Kasper C (2006). Premature menopause in survivors of childhood cancer: a report from the childhood cancer survivor study. J Natl Cancer Inst.

[CR42] Somigliana E, Terenziani M, Filippi F, Bergamini A, Martinelli F, Mangili G et al (2018) Chemotherapy-related damage to ovarian reserve in childhood cancer survivors: interpreting the evidence. J Assist Reprod Genet 10.1007/s10815-018-1345-8 PMC6420530 30362055

[CR43] Teh WT, Stern C, Chander S, Hickey M (2014). The impact of uterine radiation on subsequent fertility and pregnancy outcomes. Biomed Res Int.

[CR22] van der Kooi ALF, Brewster DH, Wood R, Nowell S, Fischbacher C, van den Heuvel-Eibrink MM (2018). Perinatal risks in female cancer survivors: a population-based analysis. PLoS ONE.

[CR25] van de Loo L, van den Berg MH, Overbeek A, van Dijk M, Damen L, Lambalk CB (2019). Uterine function, pregnancy complications, and pregnancy outcomes among female childhood cancer survivors. Fertil Steril.

[CR6] van den Berg M, van Dulmen-den BE, Overbeek A, Ronckers C, van Dorp W, Kremer L (2014). Fertility studies in female childhood cancer survivors: selecting appropriate comparison groups. Reprod Biomed Online.

[CR7] van den Berg MH, Overbeek A, Lambalk CB, Kaspers GJL, Bresters D, van den Heuvel-Eibrink MM (2018). Long-term effects of childhood cancer treatment on hormonal and ultrasound markers of ovarian reserve. Hum Reprod.

[CR44] Visser GH, Eilers PH, Elferink-Stinkens PM, Merkus HM, Wit JM (2009). New Dutch reference curves for birthweight by gestational age. Early Hum Dev.

[CR45] Wallace WH, Kelsey TW (2010). Human ovarian reserve from conception to the menopause. PLoS ONE.

[CR46] Winther JF, Boice JD, Svendsen AL, Frederiksen K, Stovall M, Olsen JH (2008). Spontaneous abortion in a Danish population-based cohort of childhood cancer survivors. J Clin Oncol.

[CR47] Wo JY, Viswanathan AN (2009). Impact of radiotherapy on fertility, pregnancy, and neonatal outcomes in female cancer patients. Int J Radiat Oncol Biol Phys.

