# Supplementary material for: Pregnancy, time to pregnancy and obstetric outcomes among female childhood cancer survivors: results of the DCOG LATER-VEVO study
Source: J Cancer Res Clin Oncol. 2020 Mar 27;146(6):1451–62. doi: 10.1007/s00432-020-03193-y (PMC7230041; doi:10.1007/s00432-020-03193-y)
Supplement: Supplementary file 1 — Supplementary file1 (DOCX 25 kb) [file 432_2020_3193_MOESM1_ESM.docx]

**Pregnancy, time to pregnancy and obstetric outcomes among female childhood cancer survivors: results of the DCOG LATER-VEVO study**

M. van Dijk^1^, F.E. van Leeuwen^2^, A. Overbeek^1,3^, C.B. Lambalk^3^, M.M. van den Heuvel-Eibrink^4^, W. van Dorp^5^, W.J. Tissing^4^, L.C. Kremer^4^, J.J. Loonen^6^, B. Versluys^4^, D. Bresters^4^, C.M. Ronckers^4^, H.J. van der Pal^4^, C.C.M. Beerendonk^7^, G.J.L. Kaspers^1,4^, E. van Dulmen-den Broeder^1,4*^, M.H. van den Berg^1*^, on behalf of the DCOG LATER-VEVO study group.

^1^Emma Children’s Hospital, Amsterdam UMC, Vrije Universiteit Amsterdam, Paediatric Oncology, Amsterdam, The Netherlands, ^2^Netherlands Cancer Institute, department of Epidemiology, Amsterdam, The Netherlands, ^3^Amsterdam UMC, Vrije Universiteit Amsterdam, department of Obstetrics and Gynaecology, Amsterdam, The Netherlands, ^4^Princess Máxima Center for Pediatric Oncology, Utrecht, The Netherlands, ^5^Erasmus MC-University Medical Centre Rotterdam, department of Gynaecology and Obstetrics, Rotterdam, The Netherlands, The Netherlands, ^6^Radboud University Medical Center, department of Haematology, Nijmegen, The Netherlands, ^7^Radboud University Medical Center, department of Obstetrics and Gynecology, Nijmegen, The Netherlands.

* Both EvD-dB and MvdB contributed equally as last authors.

Corresponding author: M. van Dijk

Amsterdam UMC, Vrije Universiteit Amsterdam

Department of Paediatric Oncology

P.O. box 7057, 1007 MB, Amsterdam

E-mail: [marloes.vandijk@amsterdamumc.nl](mailto:marloes.vandijk@amsterdamumc.nl)

**Supplementary tables**

*Supplementary Table 1. Diagnosis- and treatment-related factors associated with ever having been pregnant according to four different models: 1) type of diagnosis; 2) CED score; 3) radiotherapy site; 4) stem cell transplantation^a^.*

|  | **Number of women** | **OR (95% CI)** |
| --- | --- | --- |
| **Model 1: Type of diagnosis**  Controls  Leukaemia  Lymphoma  CNS tumours  Neuroblastoma and other peripheral nervous cell tumours  Renal tumours  Bone tumours  Soft tissue sarcoma  Germ cell tumours  Other^b^ | 422  161  93  27  37  48  42  32  24  18 | ref.  0.7 (0.4 – 1.1)  0.8 (0.4 – 1.7)  0.2 (0.1 – 0.6)  0.6 (0.2 – 1.4)  0.4 (0.2 – 0.7)  1.2 (0.4 – 4.3)  0.7 (0.2 – 2.0)  0.2 (0.1 – 0.4)  0.1 (0.1 – 0.4) |
| **Model 2: Age group at diagnosis** |  |  |
| Controls | 422 | ref. |
| < 10 | 289 | 0.6 (0.3 – 0.9) |
| ≥ 10 to < 13 | 80 | 0.4 (0.2 – 0.8) |
| ≥ 13 | 113 | 0.6 (0.3 – 1.0) |
| **Model 3: CED score^c, d^**  Controls  Zero (no alkylating agents)  <4000 mg/m^2^  ≥ 4000 - <8000 mg/m^2^  ≥ 8000 mg/m^2^ | 422  240  75  64  88 | ref.  0.7 (0.4 – 1.2)  0.6 (0.3 – 1.1)  0.7 (0.3 – 1.5)  0.7 (0.4 – 1.5) |
| **Model 4: Radiotherapy body site^e^**  Controls  No RT  Other RT only (no cranial or spinal RT, no lower abdominal/pelvic RT, no TBI)  Cranial/spinal RT  Low dose (<20 Gy)  Medium dose (20-30 Gy)  High dose (≥ 30 Gy)  Lower abdominal/pelvic RT  Low dose (<20 Gy)  Medium dose (20-30 Gy)  High dose (≥ 30 Gy)  TBI | 422  254  56  106  28  45  27  46  18  10  18  14 | ref.  0.8 (0.4 –1.4)  0.6 (0.2 – 1.5)  1.3 (0.3 – 5.9)  0.5 (0.2 – 1.3)  0.2 (0.1 – 0.6)  0.5 (0.1 – 1.6)  0.2 (0.1 – 0.8)  0.2 (0.1 – 0.5)  0.1 (0.03 – 0.5) |
| **Model 5: Stem cell transplantation**  Controls  No  Yes | 422  457  18 | ref.  0.6 (0.4 – 0.9)  0.1 (0.04 – 0.3) |

*^a^Analysed among those women who ever pursued a pregnancy. All analyses corrected for age at time of study and educational level.*

*^b^Other diagnoses include thyroid carcinomas (n=6), retinoblastomas (n=3), hepatoblastomas (n=3), nasopharyngeal carcinomas (n=3), other (n=3).*

*^c^Cyclophosphamide equivalent dose (CED) score^32^.*

*^d^Additionally corrected for treatment with gonadotoxic radiotherapy (i.e. lower abdominal/pelvic RT and/or TBI) (yes or no).*

*^e^Additionally corrected for treatment with alkylating agent therapy (yes or no).*

*Supplementary Table 2. Time to pregnancy among subgroups of participants.*

|  | **Total number of pregnancies** | **Time to pregnancy (months); Median (IQR)** | **Ratio (95% CI)^a^** |
| --- | --- | --- | --- |
| **Pregnancies by spontaneous conception** |  |  |  |
| Controls | 747 | 3.0 (1.0 – 7.0) | ref. |
| Survivors | 667 | 3.0 (1.0 – 7.0) | 1.1 (0.9 – 1.2) |
| **Pregnancies using assisted reproductive techniques** |  |  |  |
| Controls | 36 | 10.5 (2.3 – 26.3) | ref. |
| Survivors | 49 | 16.0 (3.0 – 28.0) | 1.1 (0.6 – 2.0) |
| **First pregnancies only** |  |  |  |
| Controls | 349 | 3.0 (1.0 – 8.0) | ref. |
| Survivors | 355 | 3.0 (1.0 – 9.0) | 1.1 (0.9 – 1.2) |
| **Second pregnancies only** |  |  |  |
| Controls | 257 | 3.0 (1.0 – 6.0) | ref. |
| Survivors | 243 | 3.0 (2.0 – 7.0) | 1.1 (1.0 – 1.3) |
| **Third pregnancies only** |  |  |  |
| Controls | 113 | 3.0 (1.0 – 8.0) | ref. |
| Survivors | 87 | 2.5 (1.0 – 9.0) | 1.0 (0.8 – 1.3) |

*^a^Corrected for age at time of pregnancy and educational level.*

*Supplementary Table 3. Characteristics of participating and non-participating childhood cancer survivors (CCSs).*

|  | **Participating CCSs**  **(n=1,106)** | **Non-participating CCSs**  **(n=643)** | **P *-*value** |
| --- | --- | --- | --- |
| **Age at start of study (years)**  Median (IQR) | 27.8 (11.6) | 27.2 (12.7) | 0.20 |
| **Diagnosis** |  |  | 0.23 |
| Leukemia | 394 (35.6) | 190 (31.5) |  |
| Lymphoma | 178 (16.1) | 82 (13.6) |  |
| Renal tumours | 125 (11.3) | 74 (12.3) |  |
| CNS | 114 (10.3) | 77 (12.8) |  |
| Soft tissue sarcoma | 75 (6.8) | 75 (6.9) |  |
| Bone tumours | 70 (6.3) | 50 (8.3) |  |
| Neuroblastoma and other peripheral nervous  cell tumours | 68 (6.1) | 39 (6.5) |  |
| Other | 82 (7.4) | 53 (8.8) |  |
| **Age at diagnosis (years)**  Median (IQR) | 6.4 (8.4) | 6.5 (8.1) | 0.37 |
| **Treatment** |  |  | 0.004 |
| CT-only (+/- surgery) | 554 (50.2) | 287 (45.1) |  |
| RT-only (+/- surgery) | 80 (7.2) | 59 (9.3) |  |
| CT + RT (+/- surgery) | 380 (34.4) | 209 (32.9) |  |
| Other | 90 (8.2) | 81 (12.7) |  |

*Values represent the number (%) of women, unless indicated otherwise. The subcategories may not add up to the total number of women due to missing values.*

*CNS = central nervous system; CT = chemotherapy; IQR = Interquartile range; RT = radiotherapy.*
